# Precise quantification of the time course of voluntary activation capacity following Botulinum toxin injections in the biceps brachii muscles of chronic stroke survivors

**DOI:** 10.1186/s12984-020-00716-z

**Published:** 2020-07-23

**Authors:** S. Chandra, B. Afsharipour, W. Z. Rymer, N. L. Suresh

**Affiliations:** 1 Shirley Ryan Ability Lab, 355 East Erie St., 21st floor, Chicago, IL 60611 USA; 2 Department of Physical Medicine and Rehabilitation, Northwestern University, Evanston, IL USA; 3 Department of Biomedical Engineering, University of Alberta, Edmonton, CA Canada

**Keywords:** Botulinum toxin, Stroke, Spasticity, Surface electromyography, Voluntary contraction, Quantitative measurement

## Abstract

**Background:**

Spasticity is a key motor impairment that affects many hemispheric stroke survivors. Intramuscular botulinum toxin (BT) injections are used widely to clinically manage spasticity-related symptoms in stroke survivors by chemically denervating muscle fibers from their associated motor neurons. In this study, we sought to understand how BT affects muscle activation, motor unit composition and voluntary force generating capacity over a time period of 3 months. Our purpose was to characterize the time course of functional changes in voluntary muscle activity in stroke survivors who are undergoing BT therapy as part of their physician-prescribed clinical plan.

**Method:**

Our assessment of the effects of BT was based on the quantification of surface electromyogram (sEMG) recordings in the biceps brachii (BB), an upper arm muscle and of voluntary contraction force. We report here on voluntary force and sEMG responses during isometric elbow contractions across consecutive recording sessions, spread over 12 weeks in three segments, starting with a preliminary session performed just prior to the BT injection. At predetermined time points, we conducted additional clinical assessments and we also recorded from the contralateral limbs of our stroke cohort. Eight subjects were studied for approximately 86 experimental recording sessions on both stroke-affected and contralateral sides.

**Results:**

We recorded an initial reduction in force and sEMG in all subjects, followed by a trajectory with a progressive return to baseline over a maximum of 12 weeks, although the minimum sEMG and minimum force were not always recorded at the same time point. Three participants were able to complete only one to two segments. Slope values of the sEMG-force relations were also found to vary across the different time segments. While sEMG-force slopes provide assessments of force generation capacity of the BT injected muscle, amplitude histograms from novel sEMG recordings during the voluntary tasks provide additional insights about differential actions of BT on the overall motor unit (MU) population over time.

**Conclusions:**

The results of our study indicate that there are potential short term as well as long term decrements in muscle control and activation properties after BT administration on the affected side of chronic stroke survivors. Muscle activation levels as recorded using sEMG, did not routinely return to baseline even at three months’ post injection. The concurrent clinical measures also did not follow the same time course, nor did they provide the same resolution as our experimental measures. It follows that even 12 weeks after intramuscular BT injections muscle recovery may not be complete, and may thereby contribute to pre-existing paresis.

## Background

Spasticity is a characteristic feature of upper motor neuron lesions, often induced by a hemispheric stroke. The physical signs include hypertonia (a perceived increase in resistance to externally imposed joint motion) as well as hyper-reflexia, an increase in response to muscle stretch or tendon tap. A potential contributor to hyper-reflexia in post-stroke spasticity is hyper-excitability of the associated spinal motor neurons [1]. Spasticity is believed to affect up to 40% of the chronic stroke population [2]. Over the past decade, intramuscular BT is increasingly used in clinical settings to temporarily reduce spasticity in a number of human neurologic disorders [2–4], including chronic stroke. Botulinum toxin is a metalloproteinase that induces cholinergic blockade at the presynaptic level of the neuromuscular junction. This chemo-denervation reduces hypertonia as well as hyper-reflexia by reducing global motor outflow [5]. The cholinergic blockade fades away with time [6], thus stroke survivors for whom BT is prescribed as part of their clinical spasticity management plan often receive BT every 3–4 months [7]. Long term neuromuscular changes in muscles injected with BT have been reported in animal studies, although, a systematic quantitative evaluation of short and long-term neuromuscular consequences of BT on injected muscles in humans has not been performed [8]. An initial reduction of voluntary muscle capacity is to be expected, however, quantification of the initial reduction, as well as quantification of the subsequent return to baseline values could provide insight regarding neuromuscular recovery (the time course, voluntary capacity level and neural control of muscle) subsequent to chemical denervation. This information could be used to assist in driving decisions about timing and dosage of repeated doses of BT as well as the need for other rehabilitative therapies due to the potential of further weakening paretic muscle. While BT is used to reduce spasticity, BT broadly targets motor outflow so more extensive impairment of motor function is possible.

Evaluations of BT mediated alterations in spasticity levels have largely been based on clinical assessments [9] which are at best semi-quantitative in nature, lack the necessary precision to track outcomes, and may not accurately describe the time course of BT effect on voluntary muscle activation, i.e., functional capacity. As a result, such clinical analyses do not fully address BT effects on voluntary muscle activity in the upper arm of injected stroke survivors. Several studies in humans have used joint torque as an alternative to assess both the short term and long term changes in functional capacity and neural control in various muscles after BT. Hameau et al. and Berunz et al. observed and successfully quantified the effect of BT effect on voluntary strength in lower limb in a cross-sectional study at four weeks post-injection [10, 11]. Additional electrophysiological studies were performed by Lee et al., who observed reduced values of the jaw muscle sEMG in healthy individuals at three weeks following intramuscular BT injections [12]. Hamjian and Walker reported only partial recovery of foot extensor muscle sEMG activity in healthy controls at successive intervals over the course of 3 months after the intramuscular BT in ten healthy controls based on ultrasound recordings as well as compound muscle action potential (CMAP) values [13]. A few studies have utilized electrophysiological techniques to assess changes in muscle function following BT injections in stroke survivors as well [14]. Albani et al. observed a reduction of sEMG values after BT injection in wrist flexor muscles of the stroke population at one and at 6 months post-injection [15]. Similar findings of reduced sEMG and force in biceps brachii muscle at one-month post-BT in stroke population were reported by Vinti et al. [16]. Hesse et al. found a reduction of sEMG in the soleus and tibialis anterior muscle after one month of injection amongst the majority of tested hemiparetic subjects [17]. Taken overall, these studies suggest that there are potential short-term as well as long term deficits during voluntary muscle activity following BT injections in stroke muscle. However, these studies were cross-sectional in nature and only one study provided data from both sEMG and force recordings of participants.

Accordingly, our global objective here is to use quantitative measures to accurately characterize the time course of changes in voluntary activation capacity of the biceps brachii on both sides of hemispheric stroke survivors who have received intramuscular BT on their affected side, as a part of their routine clinical care plan for spasticity. This assessment was accomplished in the usual time period between successive BT injections, 3–4 months, using force and sEMG recordings taken during varying voluntary isometric contractions. Based on the aforementioned studies, long term deficits in voluntary capacity could further weaken stroke survivors who routinely are diagnosed with paresis in addition to spasticity.

Furthermore, in related animal studies, several researchers reported BT chemo-denervation induced fiber atrophy and also a reduction in total alpha motor neuron numbers, in rats at two weeks post-injection [18, 19]. Subsequent to chemical denervation, Pamphlett reported early axonal sprouting within 2 days after the BT injection in mouse resulting from neuromuscular transmission failure [20] and this has been reported to have continued until 8 weeks by others [21]. In addition to affecting muscle contractile properties, BT injections also degrade muscle architecture, including sarcolemma structure, muscle stiffness, and muscle fiber density on histological examination, within four weeks [22]. Minamoto et al. reported reductions of joint torque by 50% after 6 months in rat tibialis anterior muscle and found that these changes were correlated with the altered muscle activity [23]. In addition, they reported a persisting sEMG deficit while the contraction force recovered after 12 weeks. Thus, unique short term and long term changes in neuronal control and muscle activity in response to intramuscular BT injections are evident in animal studies; these are also speculated but not reported in human studies. Thus we sought to address both the short term and long term changes in neural control and potential re-innervation with relevant recording and analysis techniques.

Specifically, we assessed the initial (expected) decline and subsequent return of both joint torque and sEMG values, and we determined whether both variables varied concurrently. In order to assess possible central effects, i.e., changes in muscle control in addition to peripheral alterations, we computed the slope of the relationship between the generated surface EMG and force during voluntary muscle activation, which has been shown to be disrupted in paretic stroke survivors [24]. We also sought to assess whether there were changes at the motor unit (MU) level, utilizing novel sEMG electrodes. By doing so, we are able to characterize changes in muscle activation capacity as well as assess any changes in the neural control of muscle.

To summarize our results, we found that there were systematic alterations of force and sEMG induced by BT even at 12 weeks post-injection, and we recorded consistent maximal reductions of the force and the sEMG between 2 and 4 weeks, consistent with results from animal studies. A part of this work was presented at Society for Neuroscience annual meeting, 2017.

## Methods

### Subject inclusion criteria

We have recruited and tested 8 hemiplegic stroke survivors with spasticity who were to receive botulinum toxin as part of their clinical care plan. The initial timing, dosage, and location of the injection were determined by their managing physician. All participants had sustained a single hemispheric stroke at least six months prior to experimental testing. Spasticity was assessed by a physical therapist using the Modified Ashworth Score (MAS) Subjects had to exhibit a score ≥ 1 to be included in the study. The subjects were included only if they were able to perform the required experimental tasks, attain the testing position of the hand, sit continuously during the experiments, and follow the instructions with intact auditory and visual perceptions. All participants gave informed consent via protocols approved by the Institutional Review Board under the Office for the Protection of Human Subjects at Northwestern University.

### Experimental setup

Participants were seated in a Biodex, Inc. chair with the forearm cast from elbow to the wrist and fixated to a ring-mount interface. The wrist mount was attached to a six-degrees-of-freedom force-torque sensor (ATI, Inc.: Delta ATI4007, Max force 660 N). After skin preparation, including a light skin abrasion and cleaning with alcohol pads, bipolar sEMG electrodes were placed on the medial and lateral heads of biceps brachii, triceps brachii and brachioradialis muscle (for online monitoring purposes). A pair of Delsys Inc., *sensor array* electrodes were mounted and aligned with the bipolar electrodes at a proximal location on the belly of the biceps muscle on both heads. The locations of electrodes were determined after muscle palpation on the belly of the muscle of interest, based on the recommendation of SENIAM [25]. The location of the electrodes and the load cell attachment was referenced to anatomical landmarks such as the bony prominence of the subject’s arm (lateral and medial epicondyles and acromion). SEMG recordings were performed during voluntary isometric, non-fatiguing elbow flexion force generation.

**Fig. 1:**
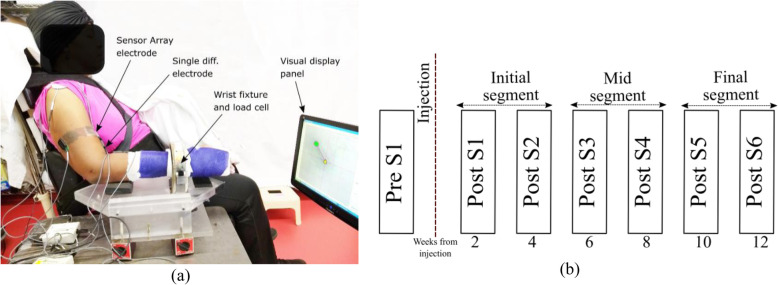
(**a**) Depiction of the experimental setup. (**b**) The time-course description of the designed experiment

A depiction of the experimental setup is shown in Fig. 1a. The EMG sensor locations were similarly maintained throughout all the sessions for all the subjects by measuring the electrode locations with respect to bony landmarks at the first pre-BT session and by documenting with photographs of the tested arm only. Signal acquisition was performed simultaneously through *Spike 2*® and the CED-1401®, and dEMG (Delsys Inc.) system decreasing force levels back to resting level. The force and the sEMG signal was sampled at a rate of 2 kHz.

### Study protocol

Clinical assessments of spasticity, the Modified Ashworth Score (MAS), and motor impairment, the Fugl-Meyer assessment (FM) were performed by a research occupational therapist at each recording session on the upper extremity on both sides of our participants. After completing the experimental recording sessions on the affected side, patients were asked to return for testing on their contralateral limbs using the same protocol.

Our protocol was designed to test the participants in one session before their respective BT injections (Pre-S1) and then 6 times after the injection (Post-S1- Post-S6), approximately every two weeks on both the affected and contralateral limbs. We have separated the observations in three segments across the time, initial segment (Post-S1-Post-S2), mid segment (Post-S3-Post-S4), and the final segment (Post-S5-PostS6) as shown in Fig. 1b. At each session, requisite submaximal force task levels were determined by asking the participants to perform maximum voluntary contractions during the session.

### Experimental protocol

Subjects were seated in an upright posture on with arm set at 120^0^ elbow flexion, 45^0^ shoulder flexion, 16^0^ shoulder abduction, and the forearm pronated to 45^0^. These angles were determined by a research therapist, to ensure maximum biceps activation, with the forearm in a mid-supine position. For consistency, we maintained the same joint angles across all recording sessions.

All subjects were instructed to produce isometric elbow flexion force in this position using visual force feedback provided by a display screen, throughout the various sessions. Two-dimensional force feedback was provided to ensure that the subjects generated pure elbow flexion force, which in the seated position results in a 45^0^ angle between the two measured force components (Force X and Force Z). A strap was used to maintain trunk and the shoulder position while subjects were performing force-producing tasks.

All subjects were asked to perform a Maximum Voluntary Contraction (MVC) at the beginning of each session. Two MVC trials were performed with 5 min of rest in between. Verbal cues were provided to the participants during the MVC trial. The higher contraction force level of the two trials was selected as the MVC value for that recording session. Subsequent force levels were set at several submaximal levels i.e. 30, 40 and 50%/ 60% of the recorded MVC. Multiple trials at each force level were performed in random order by all of our participants. A gap of two minutes between each trial was used to minimize muscle fatigue. Subjects were queried continuously regarding systemic as well as muscle fatigue. Preprocessing of force and sEMG data was performed before further analysis of RMS-sEMG (RsE)-force relations for all the force-torque levels. A commercial computational package (*Matlab®*) was used.

We matched the effort level as a percent of maximum capacity while testing submaximal force values across sessions on the affected side, as several subjects lost up to 74% of their force production capacity compared to the pre-injection session, and thus matching of absolute force levels was not possible. Varying levels of submaximal force testing was performed to understand whether similar effects were observed over the successive testing sessions and to be able to compute the relationship between sEMG and force.

### Force trajectory

**Fig. 2:**
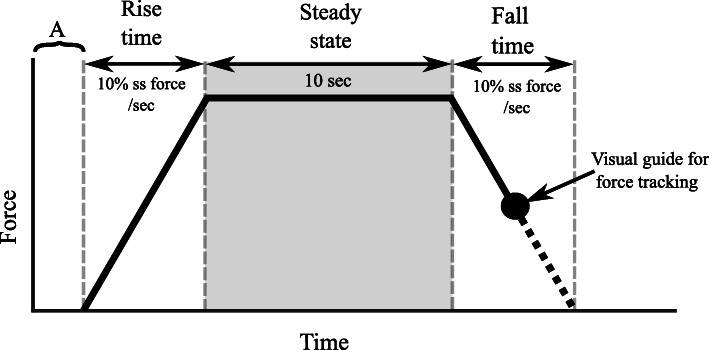
Diagram of the force trajectory guide

To increase the accuracy of the performance, a visual 2-D display of a trapezoidal force trajectory guide was provided to the subjects along with auditory feedback at the beginning and end of each force trajectory. The instructed force trajectory was programmed to control the rate of force rise. The trapezoidal force trajectory is shown in Fig. 2, starting section (marked as section A in Fig. 2) is the pretrial resting level, followed by the increasing force level to steady-state and finally the falling edge. The resultant force (F_r_) was calculated from the recorded force data from two principal axes (x, z) as:

1 $$ {F}_r=\sqrt{F_x^2+{F}_z^2} $$

F_x_ is the component of force in shoulder abduction direction and F_z_ is the component in the elbow flexion direction. The average resultant force F_r_ during steady-state force production with minimum variation (5 s) was designated as the mean force value calculation. Identical time windows of steady-state force were selected for further analysis of both force and sEMG. In the case of MVC trials, the peak value of the force was designated for further analysis and other submaximal contraction level mean force values were calculated.

### sEMG signal processing

All channels of single differential sEMG signals were initially amplified with a constant gain of 1000. Each channel data has then been offset nullified and filtered with a Butterworth FIR filter with a passband of 5 Hz to 500 Hz. Five seconds of stationary sEMG and force data were selected during the stable region of the steady-state force trace (less than 10% variation). The average signal power of the sEMG can be calculated by root mean square value of the sEMG (RsE) representing a value proportional to muscle activation capacity. The RsE was calculated within the steady-state force window as:

2 $$ RsE=\sqrt{\frac{1}{N}\sum \limits_{n=1}^N{\left|{v}_n\right|}^2} $$

**Fig. 3:**
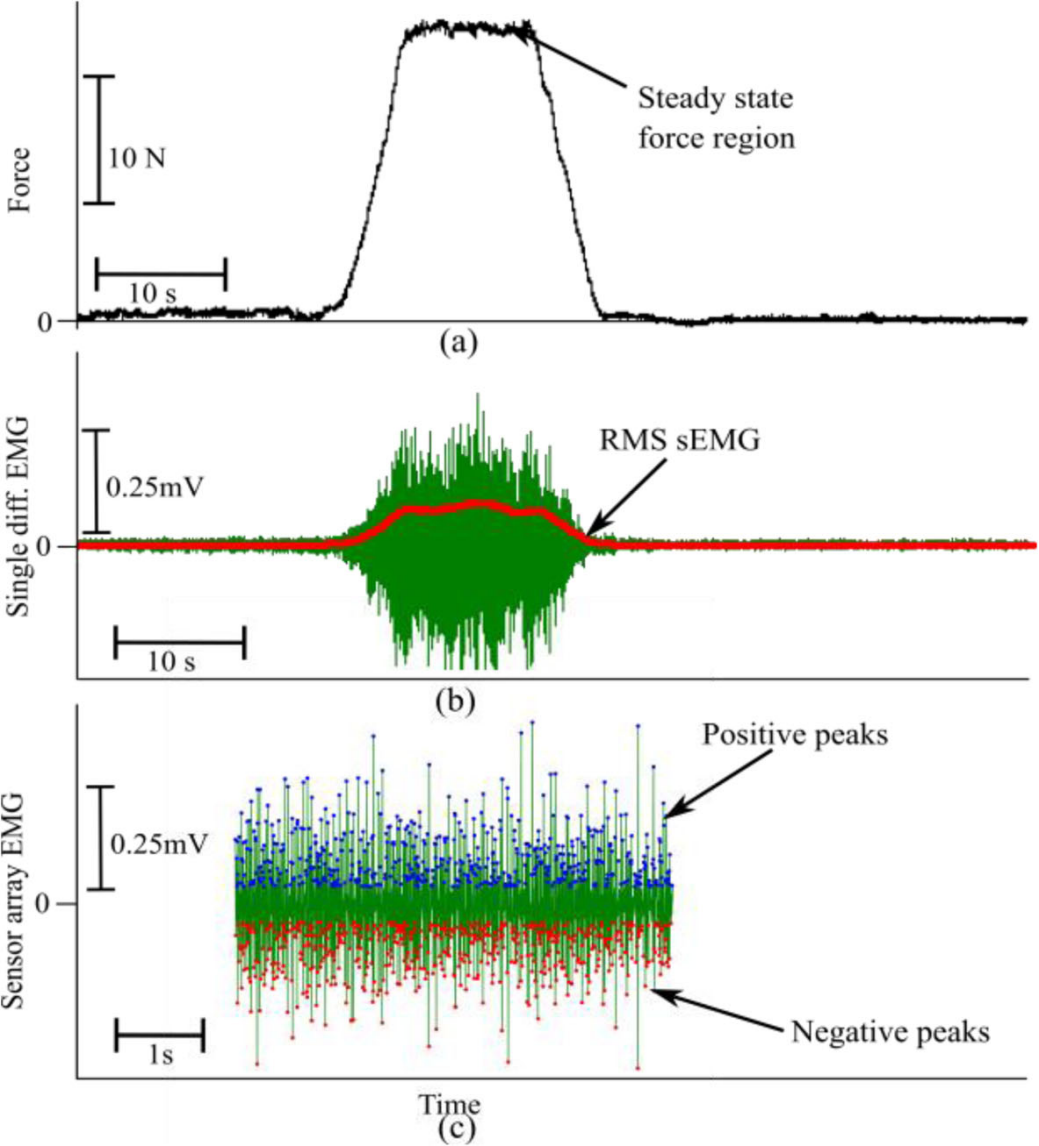
(**a**) Resultant force, (**b**) the sEMG signal from single differential electrode along with the RMS sEMG signal (in red color), and (**c**) the positive and the negative peaks from the sensor array electrode recorded from the biceps brachii muscle medial head in a time synchronous manner. The recordings depict a particular trial of B1 in the contralateral side

Where N is the number of samples within the steady-state force window and v_n_ is the absolute voltage amplitude of the n^th^ sample. These values were used for further processing as described in forthcoming sections. The percentage reduction of contraction force or RsE (*D*_*i*_) at the i^th^ session of the measurements was calculated as:

3 $$ {D}_i=\frac{M_{pre}-{M}_i}{M_{pre}}\times 100\% $$

where M_pre_ is the measurement before the injection and M_i_ is the measurement at i^th^ session after the BT injections. The correlation of the force and the RsE were analyzed with a linear regression model and all the data points were sampled at the same frequency. Figure 3a and 3b show the raw force signal and the sEMG from the medial head of the biceps muscle along with the RsE on the same time scale.

### sEMG peak detection

Surface EMG signal peaks were detected from the raw signals recorded from both heads of the biceps brachii muscle, using novel sEMG sensor array electrodes (Delsys. Inc., Boston, MA). The peaks were derived from a 5-s window in the sEMG signal during the steady-state period for the force trajectory. This electrode consists of five pins and generates four channels of differential sEMG signals. The channel with the largest peak to peak value of the raw sEMG signal was selected for the peak analysis, this was not found consistently in the same channel for every subject. The Delsys sensor array allows a higher bandwidth with reduced chances of superposition of MU spikes, making it a stronger candidate for sEMG peak detection application. The high recording sampling rates of the sensor array further support precise detection of peaks representing the MU firing instances. Figure 3c shows the sensor array recording and the detected peaks. More detailed descriptions of the sensor can be found in [24]. During data collection, the quality of sEMG recordings was checked repeatedly, as the electrode is highly sensitive to fluctuations, due to the small contact area of the sensor pins (0.5 mm diameter). Offline, the signal-to-noise ratio (SNR) was calculated, and recording trials were selected for further analysis when SNR was found to be above 15 dB. In order to select the peaks, a defined threshold was established above which all the peaks were considered for the distribution analysis. The threshold *E*_*TH*_ was defined as:

4 $$ {E}_{TH}=\mu +3\delta $$

Where μ and δ are the mean and the standard deviation of the baseline sEMG signals before the start of the voluntary activation [24]. The total number of peaks was calculated as the sum of all positive and negative peaks. Finally, the norm of both positive and negative peak values was estimated for the calculation of histogram. A Wilcoxon rank sum test was performed between the sample values to detect significant difference values obtained from the two sides of each subject and considered to be significant only of *p* < 0.05.

## Results

We report here on eight subjects who were studied before (Pre-S1) and after (Post-S1) the BT injection to assess the short term effect. Six of the eight tested subjects were able to continue testing to the mid segment (Post-S3-Post-S4), and 5 of the tested subjects continued to the final segment (until Post-S6) of the study. The subjects who could not complete the full span of the study (i.e., from the first to the third segment) were incidentally random among our cohort and were not systematically different from the participants who had continued with the total course of the study, these three participants could not complete the desired course of the study because of personal reasons (job, traveling, and adverse weather).

We collected experimental and clinical data biweekly from stroke survivors, across a maximum of 14 weeks per subject, for a total of approximately over 85 experimental recording sessions in both stroke-affected and contralateral arm muscles. Our subject cohort had not undergone physical/occupational therapy sessions, during the course of testing sessions of this study; to our knowledge, this was also not a part of their prescribed care plan. Our subject cohort did not report any pain during the sessions. The results of our experimental data collection and analysis, as well as the clinical assessments, are summarized below.

### Subject information

The subject details, injection dosages and clinical measures of the tested subjects are summarized in Table 1.

**Table 1:** Subject details, injection dosage, clinical scores before the injection and the number of weeks they were tested after the injection

Subject	Age	Gender	Post-Stroke (years)	Impaired Side	BT Dosage (human unit)		MA Scores Before Injection	FM Scores Before Injection	No. of years of BT injection (Non-continuous)	No. of weeks tested after BT
					Biceps Brachii	Brachio- radialis				
B1	56	Female	7	Right	**50**	0	1+	17	3	12
B2	40	Female	10	Left	**80**	30	1+	19	9	12
B3	60	Male	5	Right	**50**	0	2	11	5	12
B4	43	Female	4	Left	**40**	0	3	23	1	12
B5	55	Male	5	Left	**100**	0	1+	20	3	12
B6	30	Female	12	Left	**200**	0	3	14	8	6
B7	62	Male	7	Right	**50**	0	1+	19	0	2
B8	84	Male	6	left	**50**	0	3	14	5	2

### Contraction forces in affected and contralateral limbs

For all our subjects, examined across pre-injection and post-injection sessions, recorded contralateral elbow joint flexion force was considerably greater (*p* < 0.001) than the impaired elbow flexion force. Along with the force, the RsE values of the biceps brachii muscles in the contralateral side were also routinely higher (*p* < 0.05) than on the affected side.

### Tracking elbow force and sEMG (biceps brachii, triceps) during maximum voluntary contractions across all sessions on the affected side

**Fig. 4:**
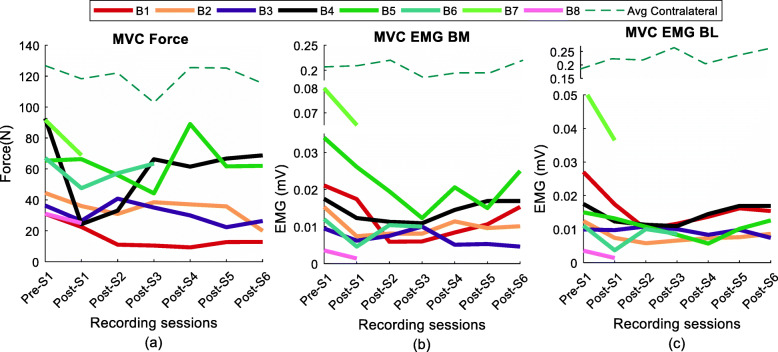
(**a**) Elbow contraction force, (**b**) Biceps medial RsE, (**c**) Biceps lateral RsE at MVC trial across the sessions for all subjects. The contralateral recording of force and the sEMG is shown with the dotted line. The contralateral RsE values for the biceps medial and biceps lateral has been plotted with a discontinuity in the vertical axis range

All tested participants show varying maximum elbow flexion force and biceps brachii sEMG values across the tested sessions, starting with a reduction in maximum force and sEMG values in the initial segments and a subsequent increase towards pre-BT recorded levels during the final segments as shown in Fig. 4a, b and c.

The initial reduction in force and sEMG occurred within 2 to 4 weeks post-injection in all tested subjects. In four out of the five subjects who had continued for the full 12 weeks, the final recorded MVC force and RsE at approximately 12 weeks post-BT, was still lower than the measured pre-BT values by approximately 25–60% (Table 2). Across the tested stroke cohort, force and sEMG signals did not exhibit the same time course.

**Table 2:** Percentage changes in elbow force, biceps medial (BM) and biceps lateral (BL) sEMG during MVC compared to the Pre -S1 session (baseline) recording. Compared to the pre-injection recording, the decreases are marked by (↓) and the increases are marked by (↑). Values are approximated to first decimal point. Dropped out sessions were denoted with NA

	Post-S1			Post-S2			Post-S3			Post-S4			Post-S5			Post-S6		
	Force	BM	BL	Force	BM	BL	Force	BM	BL	Force	BM	BL	Force	BM	BL	Force	BM	BL
B1	27.3**↓**	17.5**↓**	31.4**↓**	64.4**↓**	72**↓**	62.9**↓**	66.3**↓**	71.5**↓**	57**↓**	70.1**↓**	60.1**↓**	49.2**↓**	59**↓**	49.7**↓**	40**↓**	58.4**↓**	27.4**↓**	42.9**↓**
B2	15.4**↓**	23.2**↓**	26.6**↓**	30.4**↓**	47**↓**	53.2**↓**	13.4**↓**	47.05**↓**	46.8**↓**	16.5**↓**	25.5**↓**	41.1**↓**	19.5**↓**	37.2**↓**	38.7**↓**	55**↓**	34**↓**	30.6**↓**
B3	27.7**↓**	34.7**↓**	2**↓**	12.2**↑**	21**↓**	10**↑**	2.2**↓**	33.6**↓**	3.5**↓**	17.5**↓**	46.3**↓**	16.1**↓**	38.6**↓**	44.2**↓**	1**↓**	27.4**↓**	51.5**↓**	25.2**↓**
B4	73.9**↓**	29.7**↓**	29.7**↓**	64.3**↓**	35.4**↓**	35.4**↓**	28.5**↓**	37.7**↓**	37.7**↓**	33.6**↓**	17.1**↓**	17.1**↓**	28 **↓**	3.4**↓**	3.3**↓**	25.8**↓**	3.4**↓**	3.4**↓**
B5	1.5**↑**	23.2**↓**	12**↓**	14.2**↓**	42.6**↓**	28.6**↓**	32.3**↓**	63.8**↓**	44.6**↓**	36.3**↑**	39.4**↓**	62**↓**	5.7**↓**	55.8**↓**	33**↓**	5**↓**	26.4**↓**	16**↓**
B6	29.1**↓**	61.4**↓**	66.3**↓**	14.7**↓**	13.7**↓**	9**↓**	5.44**↓**	16.1**↓**	21**↓**	NA	NA	NA	NA	NA	NA	NA	NA	NA
B7	25**↓**	17.5**↓**	44.2**↓**	NA	NA	NA	NA	NA	NA	NA	NA	NA	NA	NA	NA	NA	NA	NA
B8	24.9**↓**	60**↓**	59**↓**	NA	NA	NA	NA	NA	NA	NA	NA	NA	NA	NA	NA	NA	NA	NA

Some similarities during the initial segment, across the tested cohort were that after the BT injection, a reduction in maximal force capacity was observed for all the subjects at the testing session approximately 2 weeks’ post-injection (Post-S1), compared to the Pre-S1 session. Correspondingly, the RsE for biceps medial and biceps lateral declined for all 8 subjects starting at the first post session (Post-S1), except for the biceps lateral recordings in B3.

However, the maximal force reduction occurred at different post-BT session. For subjects B1 and B4, the maximal force reduction of 65 and 73% (referenced to MVC force in the pre-injection) was recorded at 4 weeks and 2 weeks’ post sessions (respectively). Whereas maximal force reductions of 54, 38, and 32% were obtained for B2, B3, and B5 respectively at Post-S6, Post-S5 and Post-S3 sessions. B6, B7, and B8 also showed above 25% reduction of contraction force by Post-S1. The initial sharp reduction of MVC force (from pre-BT) values was followed by a general upward trend of MVC force values in majority of participants (B1, 2, 4, 5, 6). In contrast, a return to baseline, i.e., pre-BT values, was not recorded even at 12 weeks’ post-injection. Even at the final segment of the study (Post-S6), approximately 3 months after the injection, the MVC force was found to reach just above 60% of the force produced before the BT injection for the respective subjects. The MVC RsE of the biceps brachii muscle also showed similar trends as the force values, across sessions, however, the maximal reductions of the RsE and force did not occur at the same post-session. Reductions in the force and the RsE were calculated as a percentage difference with respect to their corresponding Pre-S1 values (baseline) using the eq. (3) (see Methods) and are summarized in Table 2.

#### Triceps muscle

We performed similar analyses on data from the *triceps* muscle on the affected side, but no systematic variation was observed in the triceps RsE across all sessions for all participants (the maximum variation across all tested sessions was within 8%).

### Force and sEMG (biceps brachii, triceps) during maximum voluntary contractions across all sessions on the contralateral side

Force and sEMG (biceps, triceps) data analyzed on the contralateral side also did not show significant systematic variation (*p* value> 0.8) with respect to the pre-BT values within a subject, however, there was variation in (absolute) values across subjects. The dotted values in Fig. 4 show the average contralateral data.

### Characteristics of varying submaximal contraction levels

**Fig. 5:**
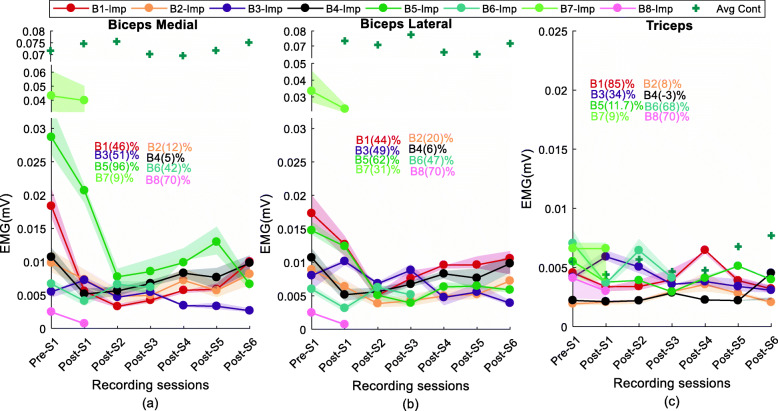
RsE values in (**a**) biceps medial, (**b**) biceps lateral, (**c**) triceps muscle during submaximal contraction. The solid line and the dots are the average value of all the responses and the shaded area indicates the variance. In parentheses, are the average percentage reduction of values at the last post-injection session compared to the pre-injection session. The average contralateral values are plotted (**+**) at a different range for biceps medial and biceps lateral with a discontinuity in the vertical axis

Along with the MVC values, multiple graded submaximal contractions were performed by all the subjects at 30, 40, and 50 (or 60%) MVC at the same session. The submaximal values were determined on the basis of the MVC recorded for that particular session. We used these data to assess the relationship between RsE and force across testing sessions, as well to assess if there were similar trends in the time course of force and sEMG at submaximal levels, as were observed at maximal levels. In Fig. 5 we plot the average of the recorded RsE values of three submaximal contractions for each tested participant across time. Percentage reductions of the RsE at the last post-injection recording compared to Pre-S1 are denoted in the figure in parentheses. The rate of decrease and recovery in RsE was different amongst the tested subjects.

The short term sharp drop of RsE was observed within the initial segment of our study. When compared individually, seven subjects (except B3) show a reduction of the RsE of the injected biceps muscle by Post-S2. A pairwise test of the consecutive recording session reveals that the values of the RsE in both medial and lateral head recording for all the subjects were significantly reduced (*p* < 0.05) compared to the pre-BT session at the second session (Post-S2). For subject B1-B5 the value remained low until the final segment (Post-S5).

#### Contralateral muscle, triceps muscle

Unlike the injected side, the RsE values in the contralateral side did not show any similar pattern with any statistical significance (*p* > 0.1). Contrary to the injected biceps muscle, no significant variation in sEMG activity was observed in triceps muscle, to within 10% across all the sessions for the subjects.

In summary, during maximal and sub-maximal contractions, the biceps brachii RsE and elbow force values recorded on the injected side of all participants exhibited a significant reduction at initial segment (2–4 weeks post-injection) followed by an incomplete return to baseline (pre-BT values) in both sEMG and force by the final segment. We observed an average 50% (for B1-B8) force reduction in the initial segment subsequent to the injection and during the final segment at the end of 12 weeks, it remained 35% lower (for B1-B5) compared to their respective baseline force values.

The sEMG during the maximal and the submaximal contraction showed a greater reduction compared to baseline (average maximum reduction > 70%) as compared to the respective force values. The reductions of the sEMG also persisted till the final segment after the injection and the value remained lower than baseline values at a statistically significant level (within 40% of the pre-injection baseline value) at the end of 12 weeks. An average reduction of 22% of RsE was observed for three subjects (B6, 7, 8) at their last recording session.

### Correlation between RsE in BM and BL

We quantitatively analyzed the correlation between recorded activity in the medial and lateral heads of the affected biceps muscle of each participant. The RsE values of the biceps medial and lateral were found to be substantially correlated (r > 0.85) in the impaired side for four subjects (B1–0.91, B2–0.93, B4–0.98, B5–0.95, B6–0.91, B7–0.95, B8–0.93) with the acceptably low *p* values (<<.05).

### RsE - force regression analysis

We quantified the relationship between the RsE values, for BM and BL separately, and recorded joint force values at submaximal contraction levels during the steady-state portion of the force trajectory (over 5 s duration) at each session for each participant. The objective was to understand if there were significant alterations in muscle control with acute chemical muscle denervation. To quantify this relationship, we regressed the computed RsE with the generated (absolute) force values with a first-order linear relationship. The correlation of the force and the RsE is summarized in Table 3. A high value of positive correlation (c > 0.75) for all the subjects was found, that represents commensurate behavior of the force and the biceps sEMG at different levels of contraction across different sessions and also ensures that the RsE-force regression slope is a representative marker that can track the variation of force and sEMG across the sessions.

**Table 3:** Correlation values among force-biceps medial sEMG and force-biceps lateral sEMG

Subject	B1	B2	B3	B4	B5	B6	B6	B7
BM-Force	0.79 ± 0.2	0.99 ± 0.005	0.77 ± 0.2	0.97 ± 0.02	0.95 ± 0.03	0.82 ± 0.01	0.88 ± 0.02	0.84 ± 0.01
BL-Force	0.80 ± 0.2	0.99 ± 0.01	0.79 ± 0.17	0.97 ±0 .03	0.97 ± 0.02	0.77 ± 0.03	0.94 ±0 .01	0.79 ±0 .05

**Fig. 6:**
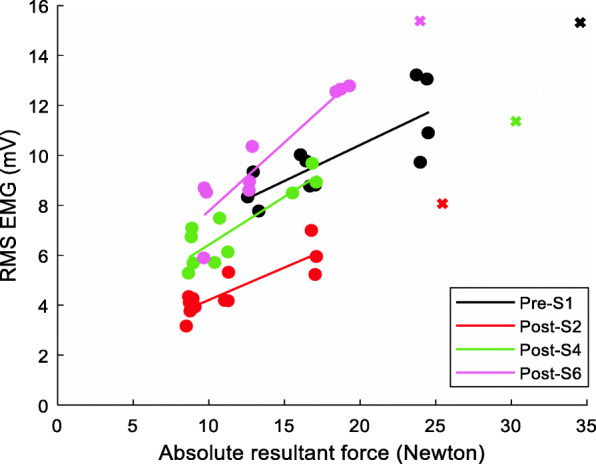
The data recorded from the subject B2 shows the linear fit at different alternate sessions in different colors as indicated in the figure legends. The MVC force sEMG points have been plotted with **×** symbol, while various sub maximal contraction forces ranging from 30% MVC to 60% MVC have been indicated with ● symbol

The computed slope values of the regression analysis across various recording sessions were further analyzed to assess any consistent trends as a function of time after BT injection. A representation of the slope of RsE-force fit during various submaximal contraction levels across all recording sessions for a subject is shown in Fig. 6.

**Fig. 7:**
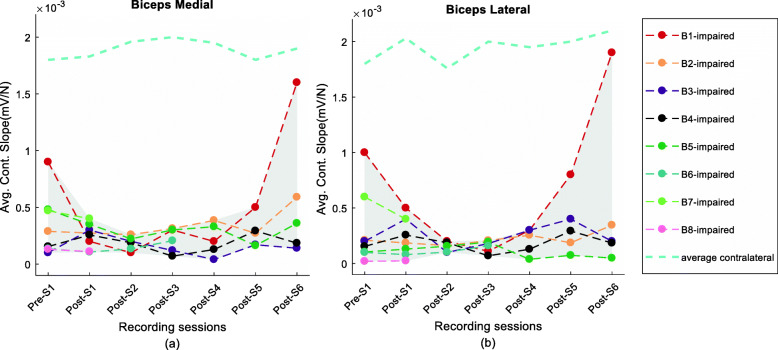
RsE-force slope values in (**a**) biceps medial and (**b**) biceps lateral

Summary plots of the Biceps RsE-force slopes for each subject across all experimental sessions are shown in Fig. 7a (BM) and 7b (BL). Seven subjects (except B5) showed a reduction of the RsE-force slope value after the injection within the initial/mid segment of the study. The slope values subsequently trended upward after the decrease and achieved a higher value during the Post-S6 session compared to their respective pre-injection level for four subjects in both biceps medial (71 ± 36) and biceps lateral (65 ± 38) recordings in all but one participant (B5) (Fig. 7a, b).

The RsE-force slope of the contralateral was found to be significantly higher than the slope on the affected side (p < < 0.05) for all subjects across all sessions, the average *contralateral* slope values were steady within 8% of the baseline across the sessions, as depicted by the blue dashed line in Fig. 7.

#### Triceps muscle

The slope value for the triceps RsE-force relation was found to be similar for both the affected and the contralateral side and the slope remained consistently flat (within 2%) across all participants, and unchanged across all sessions.

The slope values of the normalized RsE (normalized to RsE computed from data recorded during the MVC task at each session) and the force were also analyzed. This analysis was performed to better understand the percentage of the available MU pool that was utilized to generate the respective force. During the final segment (at the Post-S6 session), all the remaining subjects (except B5) showed higher slope values at the Post-S6 session compared to the respective pre-injection values in their biceps medial (B1–136%, B2–203%, B3–275%, B4–49%) and lateral (B1–267%, B2–137%, B3–12%, B4–18%). B6, B7, and B8 slope were found to be lower (< 70%) than the baseline at their last recording. Despite variability in the time course, there was an increasing trend of the values with time in the stroke-affected side. **Contralateral biceps:** The respective slope values on the contralateral side remained relatively unchanged (within 5%) across all experimental sessions.

In summary, the slope values exhibited several general trends. After attaining global minima at 2–4 weeks following the BT injection, the slope values increased in successive sessions until Post-S6, where the values were higher on average, by 165% in the biceps medial and 108% in the biceps lateral as compared to baseline i.e., pre-BT slope values in four of our five subjects that were tested at the final segment.

### Distribution of sEMG amplitude peaks

**Fig. 8:**
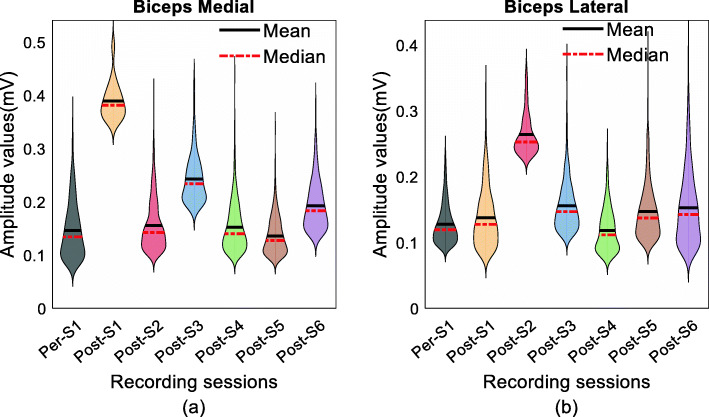
Distribution of the sEMG amplitude peaks in (**a**) biceps medial and (**b**) biceps lateral across the recording sessions for the high contraction trials for B3

An sEMG peak analysis from seven of our tested participants was performed (subject B4 was excluded as the sensor array recording was found corrupted by noise during recording). To provide insight as to the variations in RsE-force slope across time and as well to understand potential alterations in motor unit size distribution as a function of time post-BT, this analysis involved replotting the data as an amplitude histogram. All the peaks of the sEMG were identified and fit with a kernel distribution to preserve the versatility of the distribution shape as attributed by individual peak values. Figure 8 shows an example of sEMG peak distributions from medial (Fig. 8a) and lateral (Fig. 8b) heads of the injected arm of subject B3 recorded during a high range (50% MVC) contraction levels. The amplitude range of the peaks is shown along the vertical axis of the plot.

The median peak values (MPV) during the 50%MVC contraction level were compared across all recorded sessions for all 7 out of the 8 participants. In six out of the seven subjects, (B1, 2, 3, 6, 7, 8 in biceps medial, and B2, 3, 5, 6, 7, 8 in biceps lateral), the (median) peak distribution shifted (*p* < 0.001) towards the larger values in weeks 2–4 post (as compared to pre-BT values) despite the fact that during these weeks, the maximum voluntary force and maximum sEMG values were at their lowest recorded values.

#### Contralateral biceps

A similar shift of the peak amplitude distribution was not observed and the distribution (range and shape) remained unchanged throughout all the recording sessions on the contralatera*l* side (*p* ≈ 0.7).

**Fig. 9:**
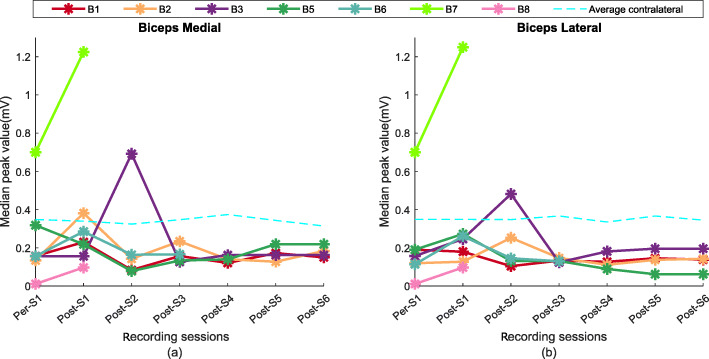
Median peak values detected in (**a**) biceps medial and (**b**) biceps lateral for the subjects across various recording session for the high contraction trial

In Fig. 9a we show a plot of the median of the amplitude histogram for all of our tested subjects. In this plot, we see that that the shift of the peak values was different among the subjects, the ratio of the highest and the lowest value of the MPV in BM recording was 4.01 ± 0.4 (B 1, 2, 3, 6, 7, 8). The biceps lateral recording shows (Fig. 9b) a similar increase of the MPV after the injection within initial segment (Post-S1 and S2) for six subjects (B 2, 3, 5, 6, 7, 8). The ratio the highest and the lowest value of the MPV in biceps lateral is 3.02 ± 0.5. While the appearance of the larger median peak value was evident among all the subjects in the injected side, no significant deviation of the MPV values found in the contralateral side (as shown in Fig. 9a, b), and remained within 10% of variation compared to its baseline values.

In summary, larger sEMG peak values were evident in the injected muscle for all the subjects within two/four weeks after the BT injection. The peak amplitude histogram median values subsequently return to baseline values within two sessions in all subjects and remain within 20% of the pre-injection baseline values. The variability of the median values on the contralateral side of the tested cohort remained under 10% across all tested sessions.

### Clinical assessment scores

**Fig. 10:**
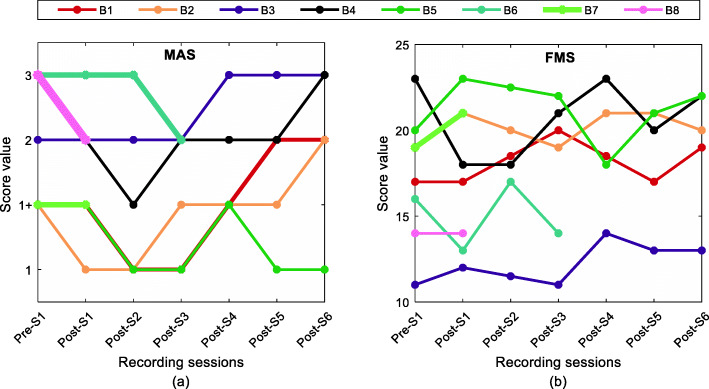
(**a**) MA score and (**b**) FM score for all the subjects across the different recording sessions

Modified Ashworth scores (MAS) and Fugl-Meyer scores (FMS) for the upper extremity were recorded at similar time points (as experimental recordings) to assess both the level of spasticity and the degree of impairment of the affected limbs. The values for MAS and the FMS are plotted in Fig. 10a and b for all subjects. The FMS showed a different pattern of variation compared to MAS. Six out of eight subjects exhibited reductions in MAS values in weeks 2–6 post-BT injection with a recovery to pre-injection levels or higher. Only subject B5 exhibited a decrease in the MAS score at the Post-S6. In four of the subjects (B1,2,3,5) higher FMS scores were evident at Post-S6 compared to the pre-BT sessions, however, within a subject, there was considerable variation across time at the tested sessions. Only three subjects (B1, 2, 5) exhibited an initial increase in the FMS scores that were correlated with the timing of their recorded decreases in the MAS scores.

## Discussion

The primary objective of this study was to characterize the time course of the effects of botulinum toxin on voluntary muscle activation capacity in stroke survivors with spasticity. We have studied the changes over three segments across the 12 weeks to precisely quantify the effect of BT on the voluntary capacity and the muscle control. Our analysis was based jointly on the myoelectric activity of the BB muscle and the associated joint force during voluntary isometric contractions after a single intramuscular BT injection, as prescribed by the participant’s clinician as part of the clinical plan of care. Clinical scores along with the elbow contraction force and the sEMG of the BB muscle have also been analyzed to understand the variation in the functional deficit of the voluntary force production capacity. Our objective was to understand if voluntary capacity and muscle function exhibited an initial (expected) reduction, followed by a sustained reduction during the final segment, and whether force and sEMG returned to baseline levels within the tracking period. We expected that the return to pre-BT levels would occur within 3 months and that it would be back at least within 10–15% of the pre-injection values.

Based on the experimental results we have found that the decrement and subsequent increases in our measured parameters, after the injection across the heterogeneous stroke cohort, are indeed different in some aspects, however, there are also similarities. As expected, the clinical assessments were lacking the necessary resolution to capture the same characteristics across time.

### The difference in clinical scores (MAS and FMS) and RsE

One of novel results of our study is that the time course of changes in the clinical scores for spasticity, i.e., the MA score, and motor impairment, i.e., the Fugl-Meyer score differs from the time course of changes in their respective force and the RsE values. Though MAS is a well-accepted measure for spasticity estimation, a measure used to determine the necessity for repeated BT injections in stroke survivors, the rating suffers major reliability issues, as addressed earlier [26, 27]. During our current study, clinical assessments were done at repeated time points; the same clinician performed the clinical evaluation throughout the study, to minimize inter-rater variability [26]. Marginal improvement of MAS and FMS within weeks after BT injection have been reported in a few case studies earlier [28, 29].

There was a lack of significant correlation between the max RsE value and the MAS values for all tested subjects. A decrement of the MAS values after BT injection has been reported earlier by other research groups and attributed to the unavailability of the fibers with active NMJ innervation [30, 31]. Despite the fact that primary actions of BT are centered around the NMJ of the injected muscle, the recovery of motor outflow (measured by RsE values during the voluntary contraction) lags the recovery of MAS, which is likely an issue due to the lack of resolution of the MAS. The other issue to consider is that the MA assessment can confound non-spastic hypertonia (i.e., due to passive muscle properties) with spastic hypertonia, in which case the reduction in motor outflow as assessed by sEMG quantification, may be imperceptible to the clinician. Factors such as acute changes in muscle architecture [32], muscle stiffness [33], following BT injections, as reported in animal studies (46), may also play an important role.

The FMS also exhibited large variability across time. We expect that BT alone cannot greatly improve functional recovery but recovery may improve with the associated occupational therapy [34], however as reported above in the Results section, none of our tested cohort was prescribed rehabilitation therapy or were enrolled in rehabilitation therapy post-BT injection. It is thus evident that the clinical scores (MAS and FMS) may be a valuable primary screening tool, but are not an accurate representation of the time course of change in muscle activation as quantified with higher resolution measures such as force and sEMG recordings after the BT therapy. Our findings are also consistent with earlier studies [30, 35].

### Differences in the time course of force and RsE recovery

In all tested participants, recorded sEMG and voluntary force signals exhibited a systematic decrement in magnitude, as expected within the initial segment of our study in the first few weeks post-BT, with respect to baseline levels (i.e., pre-BT levels). Subjects B1-B6 exhibited increasing trend back towards baseline levels in the subsequent trials. However, there was a disproportionate change in contraction force levels and RsE magnitude after the injection. As well, RsE values did not return back to pre-injection baseline values at the end of 12 weeks for the tested subjects. Submaximal force testing was performed to understand whether similar effects were observed over the successive testing sessions and to be able to compute the relationship between sEMG and force. Reduction of contractile force and of the RsE after the intramuscular injection is assumed to be primarily attributable to the reduced active muscle fiber population, which can be explained by the BT induced presynaptic blockade of the cholinergic transmission [6, 36]. Faster recovery of force may be due to altered load sharing activity from synergistic muscles during flexion [31, 37], thus our force measurement is potentially impacted by actions of other synergistic flexors. Whereas, the RsE values reflect injected muscle characteristics.

Another possible reason is the potential retrograde transportation of BT to other fibers of the muscle and to other muscles through adjacent motor neurons [6], that may produce regionally different activity in the muscle. In this study, we did not find any abnormal antagonist co-activation. The triceps muscles stayed relatively dormant across all recording sessions for all of the subjects at all contraction levels. There was an enduring deficit of motor outflow at the end of 12 weeks in the injected muscle, indicating a compromised recovery of extrafusal fibers that may accumulate further [23] resulting in a permanent reduction in the number of active muscle fibers available in long run. While there can be variability in sEMG recording over time even in a stable (un-injected) muscle, the patterns observed in the affected and injected biceps brachii muscle were not observed in the affected or contralateral triceps muscle, nor in the contralateral biceps brachii, thus we believe the measured reductions in the affected muscle to be an accurate representation of the muscle state.

The heterogeneity within and between patients was evident in both the affected and the contralateral side as recorded during the experiment. These could potentially be associated with multiple factors i.e. altered contracture in the muscle, architectural changes in the muscle (and varying levels of the injected toxin). Some of these effects have peripheral origin but some of the factors are mediated from upper motor neurons that may have produce ipsilateral deficiencies also [38].

### RsE-force slope variation

Besides analyzing the deficit in voluntary force production, another focus of this study was to investigate changes in motoneuronal control of muscle force production subsequent to the BT injection. Previous studies have reported that in chronic stroke survivors, the slope of the relation between the RsE and force was higher on the stroke affected side compared to the contralateral side potentially due to reductions in motor unit firing rates and thereby increase in the recruited motor units at a given force level in both upper limb and hand muscle [39]. Similarly, while investigating the difference in sEMG characteristics in stroke, sEMG signal peaks were further reported to be skewed toward the larger amplitude in paretic muscle [24]. None of these quantifiable measures of paresis have been used so far to identify the influence of BT on neuromuscular control in spastic individuals during voluntary contraction.

Our data shows an increased slope value during the final segment of the study (Post-S6) while the force and the sEMG were lower than the pre-injection baseline for subjects B1-B5. This suggests an increased drive to generate a similar amount of force potentially due to decremental changes in muscle structure and function.

It is possible that the slightly higher slopes are indicative of inefficient force production due to an increase in connective tissue deposits, alterations in muscle fiber size or alterations in control due to reinnervation, as reported in animal studies [18–21]. As mentioned in our results, computed slope values of RsE normalized to the max recordable RsE (at each respective session) as a function of force also exhibited significant increases in the computed slope value at the final session in all tested subjects. A normalized sEMG is less representative of decreases in sEMG due to muscle fiber inactivation in post-BT sessions, thus the greater slope suggests that there is a greater amount of recruited muscle fibers amongst the available pool for an incremental force change. A greater amount of normalized RsE for a given amount of force could arise due to sequential recruitment of larger motor units or could arise due to an increase in neural drive to compensate for the loss of muscle fiber or loss of force generating capacity.

The slope variations reported in our tested subjects cannot be attributed to varying levels of triceps co-contraction, as the triceps activation levels remained relatively constant across the testing sessions. Antagonist recordings during isometric force generation in stroke subjects in other studies [39] have also routinely shown minimal antagonist coactivation on the affected side.

### Change in the distribution of sEMG peak values

Here, we use the amplitude distribution of the sEMG peak values as a surrogate for the amplitude distribution of the MU pool in the muscle, due to the use of sensor array electrodes, which are very selective electrodes designed to record MU action potential shapes. Our reported shift of the peak values after the BT injection is potentially a reflection of the selective action of BT on the overall MU pool. The median amplitude of the sEMG peak values was increased (in six of seven subjects) despite the reduction in the values of both the RsE and force. The highest median value of the sEMG peak amplitude was found within the initial segment at the second post-injection recording session.

The shift in the amplitude of the peaks also can provide an idea about the functional properties of the detectable active motor unit population. The appearance of larger peaks may be attributed by the early terminal and nodal sprouting of motor axons in nonfunctional end plates that begin to form within days after the injection [20]. There is also a chance that the motor units created will have smaller numbers of active muscle fibers and hence a different amplitude distribution observed at a smaller force.

With time, the sprouting is inhibited but this process is comparatively slower [40], appearance and existence of the long tail with the reappearance of smaller peaks in the amplitude histogram may be explained by this phenomenon. The modified motor units after the sprouting contribute to the force generation likely become inefficient while the smaller number of the available fibers contributes to an overall reduction of sEMG even as the peak values are higher. Also, a reduced number of available fibers may enforce a different combination of recruited MU during the contraction. Eventually, the more fiber gets reconnected with the original parent axon and more muscle fibers become available, so the functionality of the MU is restored partially as observed during the final segment of our study.

There is ample evidence that BT induces progressive loss of contractile fibers [41] and induces significant atrophy of the muscle fiber [42]. Subject B7 who was injected for the first time shows highest sEMG and largest amplitude of peaks in the sEMG. This means that repeated doses of BT based on clinical scores may be detrimental to the overall contractile function of the muscle [23]. A fresh BT injection planned depending upon the MAS score alone could thus be detrimental to the muscle fibers and perhaps should be delayed further. A reduction in maximum force an sEMG that is typically recorded at the end of the three months is at least partly a reflection of muscle fiber loss or fiber which may be non-recoverable, at least in part.

## Conclusion and limitations

This study provides a quantitative characterization of functional deficits and motor outflow changes after intramuscular BT injections in stroke survivors, which allows us to more clearly understand the effects of BT on voluntary motor capacity. The results suggest the potential need to weigh the benefits of BT therapy for reduction of spasticity with the potential risk of an increase in muscle weakness. The results of this study also show that quantitative measures of force and sEMG do not track with clinical assessments, suggesting that greater measurement precision could provide improved guidance regarding BT injections for spasticity management in stroke survivors.

The broad applicability of our results are limited due to the modest number of subjects that we report on. As a function of the variable nature of the chemical denervation and subsequent reinnervation process, the time course of changes in voluntary capacity differed amongst the participants. Part of this variability could also be due to factors that were not under our control, including the dose of the injected BT and the prior history of BT injections in each participant. Thus, it is noted that to utilize these results to guide practice in a clinical setting will require a bigger participant cohort.

The variable nature of the denervation/reinnervation process combined with the numerous testing time points employed in this study could contribute to some of the differences reported across participants, i.e., the more frequent testing time points revealed greater variability across the participants. Indeed, there are striking similarities in the sEMG and force parameters observed amongst the majority of the subjects at the very early weeks post-BT injection, when most of the denervation process would have occurred and at the later weeks, when a majority of the reinnervation process may have completed. These similarities include the dramatic reduction of RsE and force in the first 2–4 weeks, with only partial recovery of RsE values at 10–12 weeks, higher force-sEMG slopes after 12 weeks and the appearance of large sEMG peaks in the peak amplitude distribution within 2–4 weeks. It is possible that in the intervening weeks, there was a fluid set of denervated /reinnervated neuromuscular junctions, based on the data reported in this study. These variations of the sEMG values were statistically tested to be significant (we have achieved an effect size of 1.4 at a confidence level of 0.05 with our test cohort), thus our preference was not to report our results as a series of case reports.

## Data Availability

The datasets used and/or analyzed during the current study are available from the corresponding author on reasonable request.

## Abbreviations

BT: Botulinum toxin-A
MAS: Modified Ashworth Score
FMS: Fugl-Meyer Score
sEMG: Surface electromyography
EMG: Electromyography
BM: Biceps medial
BL: Biceps lateral
RsE: Root mean square surface electromyography
MU: Motor Unit
MN: Motor Neuron
SNR: Signal to Noise Ratio
MVC: Maximal Voluntary Contraction
MPV: Median of Peak Values
